# Determination of *NUDT15* variants by targeted sequencing can identify compound heterozygosity in pediatric acute lymphoblastic leukemia patients

**DOI:** 10.1038/s41598-020-71468-y

**Published:** 2020-09-01

**Authors:** Chih-Hsiang Yu, Ya-Hsuan Chang, Der-Shiun Wang, Shiann-Tarng Jou, Chien-Yu Lin, Kai-Hsin Lin, Meng-Yao Lu, Lovely Raghav, Hsiu-Hao Chang, Kang-Hsi Wu, Shu-Wei Chou, Yu-Ling Ni, Dong-Tsamn Lin, Shu-Wha Lin, Hsuan-Yu Chen, Yung-Li Yang

**Affiliations:** 1 Departments of Clinical Laboratory Sciences and Medical Biotechnology, National Taiwan University, Taipei, Taiwan; 2 Institute of Statistical Science Academia Sinica, Taipei, Taiwan; 3 Department of Pediatrics, Tri-Service General Hospital, Taipei, Taiwan; 4 Graduate Institute of Clinical Medicine, College of Medicine, National Taiwan University, Taipei, Taiwan; 5 Department of Pediatrics, National Taiwan University Hospital and National Taiwan University College of Medicine, Taipei, Taiwan; 6 Division of Pediatric Hematology and Oncology, China Medical University Children’s Hospital, Taichung, Taiwan; 7 Department of Laboratory Medicine, National Taiwan University Hospital and National Taiwan University College of Medicine, No. 7, Chung Shan S. Rd, Zhongzheng Dist, Taipei City, 10041 Taiwan

**Keywords:** Haplotypes, Paediatric cancer

## Abstract

Mercaptopurine intolerance is an adverse effect of mercaptopurine administration in pediatric acute lymphoblastic leukemia. Recently, *NUDT15* variants were identified as a major determinant of mercaptopurine intolerance. Two *NUDT15* variants, c.36_37insGGAGTC and c.415C > T, are located on exons 1 and 3, respectively. Patients with heterozygous c.36_37insGGAGTC and c.415C > T can be either compound heterozygous with two variants on different alleles or heterozygous with both variants on the same allele. Because patients with biallelic *NUDT15* variants are extremely sensitive to mercaptopurine, clinical identification of *NUDT15* diplotype would be advantageous. A cohort of 37 patients with c.36_37insGGAGTC and c.415C > T *NUDT15* variants were selected for haplotyping by targeted sequencing. *NUDT15* complementary DNA was amplified and sequenced by 300-bp paired-end sequencing on Illumina MiSeq. Of the 37 patients carrying *NUDT15* variants, 35 had heterozygous NUDT15*1/*2 variants and two had compound heterozygous *NUDT1*5*3/*6 and NUDT15*2/*7 variants. These two patients with compound heterozygous variants could only tolerate low doses of mercaptopurine, similar to patients with homozygous *NUDT15* variants. Targeted sequencing of *NUDT15* cDNA can be used to determine *NUDT15* diplotype and identify patients with compound heterozygous *NUDT15* variants*.*

## Introduction

Thiopurines (e.g., 6-mercaptopurine [6-MP], 6-thioguanine [6-TG], and azathioprine [AZA]) are important antimetabolite drugs that are used for different clinical indications. In pediatric acute lymphoblastic leukemia (ALL), 6-MP is widely used in the induction, consolidation (with high-dose methotrexate), and maintenance phases. Indeed, 6-MP-based maintenance therapy is one of the most critical components in the treatment regimen for pediatric ALL^1–7^. Variants of genes responsible for thiopurine metabolism can directly influence drug toxicity and antileukemic efficacy^8–13^. For example, polymorphisms in the thiopurine methyltransferase (*TPMT*) gene have been linked to susceptibility to thiopurine-induced marrow suppression in patients, and preemptive *TPMT* genotype-guided dosing represents a successful example of genetics-based precision medicine in cancer treatment^14,15^.

**Figure 1:**
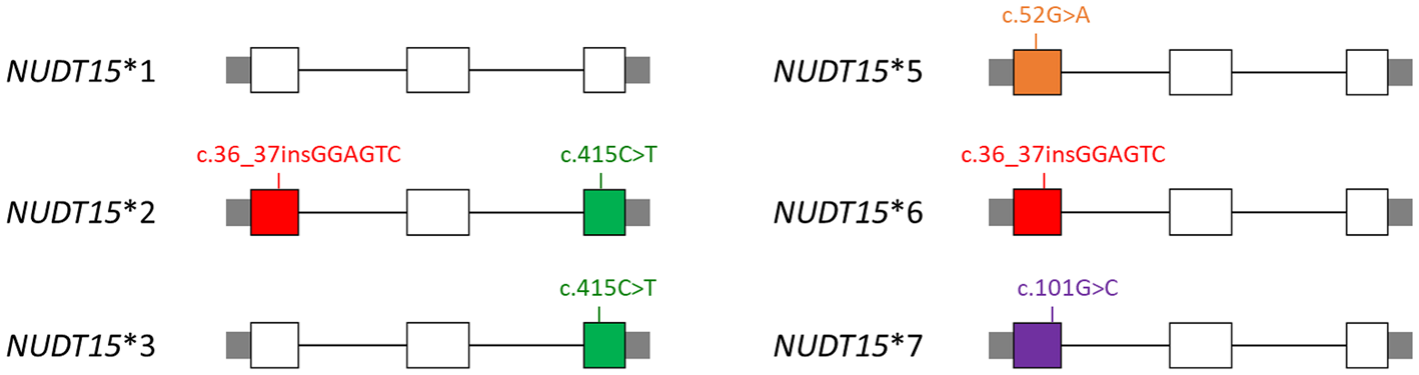
Four coding variants in *NUDT15* were identified, representing six haplotypes (labeled as NUDT15*1–3 and NUDT15*5–7).

Recent genome-wide association studies have reported a missense variant in *NUDT15* (*rs116855232*, referred to as c.415C > T or p.Arg139Cys) that is strongly associated with thiopurine-related myelosuppression in patients with inflammatory bowel disease in a Korean population^16^. In a genome-wide association study, Yang et al. identified this variant in association with mercaptopurine intolerance in childhood ALL^17^. Several *NUDT15* variants associated with decreased diphosphatase activity have been reported, and haplotypes with different combinations of the variant(s) have been assigned star allele numbers, which were proposed by Moriyama et al. (Fig. 1)^18,19^. All haplotypes except for NUDT15*2 have been reported to carry a single coding variant, including NUDT15*3 (*rs116855232,* c.415C > T), NUDT15*5 (*rs186364861,* c.52G > A), NUDT15*6 (*rs869320766,* c.36_37insGGAGTC), and NUDT15*7 (*rs766023281,* c.101G > C). NUDT15*2 carries both c.415C > T and c.36_37insGGAGTC, which are also present in NUDT15*3 and NUDT15*6, respectively. Numerous studies have established that *NUDT15* variants are associated with mercaptopurine intolerance in pediatric ALL in different populations, highlighting the importance of preemptive genetic typing in these patients^20–30^.

Because of the strong correlation between the number of *NUDT15* risk alleles and median 6-MP dosage, accurate determination of diplotypes might allow preemptive dosing^31^. For example, patients with heterozygous c.415C > T and c.36_37insGGAGTC can be determined to be either heterozygous (NUDT15*1/*2) or compound heterozygous (NUDT15*3/*6), and the tolerated 6-MP dosage by patients with the two diplotypes might be significantly different. In this study, we elucidated the *NUDT15* diplotypes in patients with multiple heterozygous variants by next-generation sequencing (NGS) of *NUDT15* complementary deoxyribonucleic acid (cDNA). Thus, this study will expand our knowledge of *NUDT15* variants in patients with pediatric ALL in Taiwan.

## Methods

### Patients

We selected pediatric patients with ALL younger than 18 years of age treated in the National Taiwan University Hospital, Taipei, between April 1997 and December 2019. This study was approved by the Institutional Review Board of the National Taiwan University Hospital. Informed consent was obtained from the parents or legal guardians of the patients. This cohort was previously published^30^, and we selected a subset of patients for this study. There were 22 patients with heterozygous c.36_37insGGAGTC and c.415C > T variants, and 10 patients with known *NUDT15* diplotypes (4 with NUDT15*1/*1, 2 with NUDT15*1/*3, 1 with NUDT15*1/*5, 1 with NUDT15*1/*6, and 2 with NUDT15*2/*2) were enrolled in the previous study^30^. In this study, another 15 patients with heterozygous c.36_37insGGAGTC and c.415C > T variants, 1 patient with NUDT15*1/*5, and 1 patient with NUDT15*2/*3 were enrolled.

### Targeted sequencing-based haplotyping

Forty-nine patient samples were selected for haplotyping by targeted sequencing, including 37 patients with heterozygous c.36_37insGGAGTC and c.415C > T variants. Samples from 12 patients with known *NUDT15* diplotypes (NUDT15*1/*1, NUDT15*1/*3, NUDT15*1/*5, NUDT15*1/*6, NUDT15*2/*2, and NUDT15*2/*3) were included as experimental controls. Total RNA was extracted using TRIzol reagent according to the manufacturer’s instructions (Invitrogen, Waltham, MA, USA). We synthesized cDNA from germline total RNA using the Maxima First-Strand cDNA Synthesis Kit (Thermo Fisher Scientific, Waltham, MA, USA). *NUDT15* cDNA was amplified using the Phusion Hot Start II High-Fidelity PCR Master Mix (Thermo Fisher Scientific) with barcoded primers (Tables S1 and S2). The barcoded amplicons were purified using AMPure XP beads (Beckman Coulter, Brea, CA, USA) and quantified using Qubit reagent (Thermo Fisher Scientific). Equimolar amounts of each amplicon were pooled and sequenced by 300-bp paired-end sequencing using the MiSeq Reagent Kit v3 (Illumina, San Diego, CA, USA). Raw sequencing data were further analyzed to discriminate the *NUDT15* haplotypes. The barcoded primers used for the targeted sequencing of cDNA are listed in Supplementary Table 1.

FastQC (v0.11.7) was performed to evaluate the quality and adaptor contamination of the sequencing reads. Adaptor sequences and low-quality bases were trimmed (average quality score of a four-base wide sliding window < 20) using Trimmomatic (v0.33). The trimmed reads were further aligned to *NUDT15* cDNA (NM_018283) using bowtie2 (v2.3.3.1). The average insert length of the reads was 562 bp. Reads with specific variants were extracted from the total aligned reads and used to determine the two alleles of *NUDT15*. Finally, IGV tool (v2.7.2) was used to check the read alignment results and the two alleles of the samples.

### Cloning of NUDT15 cDNA

The *NUDT15* cDNA amplicons were purified using the FavorPrep GEL/PCR Purification Kit (Favorgen Biotech, Ping-Tung, Taiwan), cloned into a vector using the Zero Blunt TOPO PCR Cloning Kit (Thermo Fisher Scientific), and transformed into TOP10 competent cells (Thermo Fisher Scientific). Single colonies were cultured in LB broth (50 µg/mL kanamycin) at 37 °C for 16 h, and plasmids were extracted using the FavorPrep Plasmid DNA Extraction Mini Kit (Favorgen Biotech). Sanger sequencing was performed using the SP6 or T7 primer (Supplementary Table 1). At least 20 plasmids from a single colony were sequenced to determine the *NUDT15* diplotypes.

### Statistical analyses

Mann–Whitney U test was used to evaluate differences in median 6-MP dosages between patients with a heterozygous variant (NUDT15*1/*2) and homozygous/compound heterozygous variant (NUDT15*2/*2, NUDT15*2/*3, NUDT15*2/*7, and NUDT15*3/*6).

## Results

### Next-generation sequencing of cDNA

**Figure 2:**
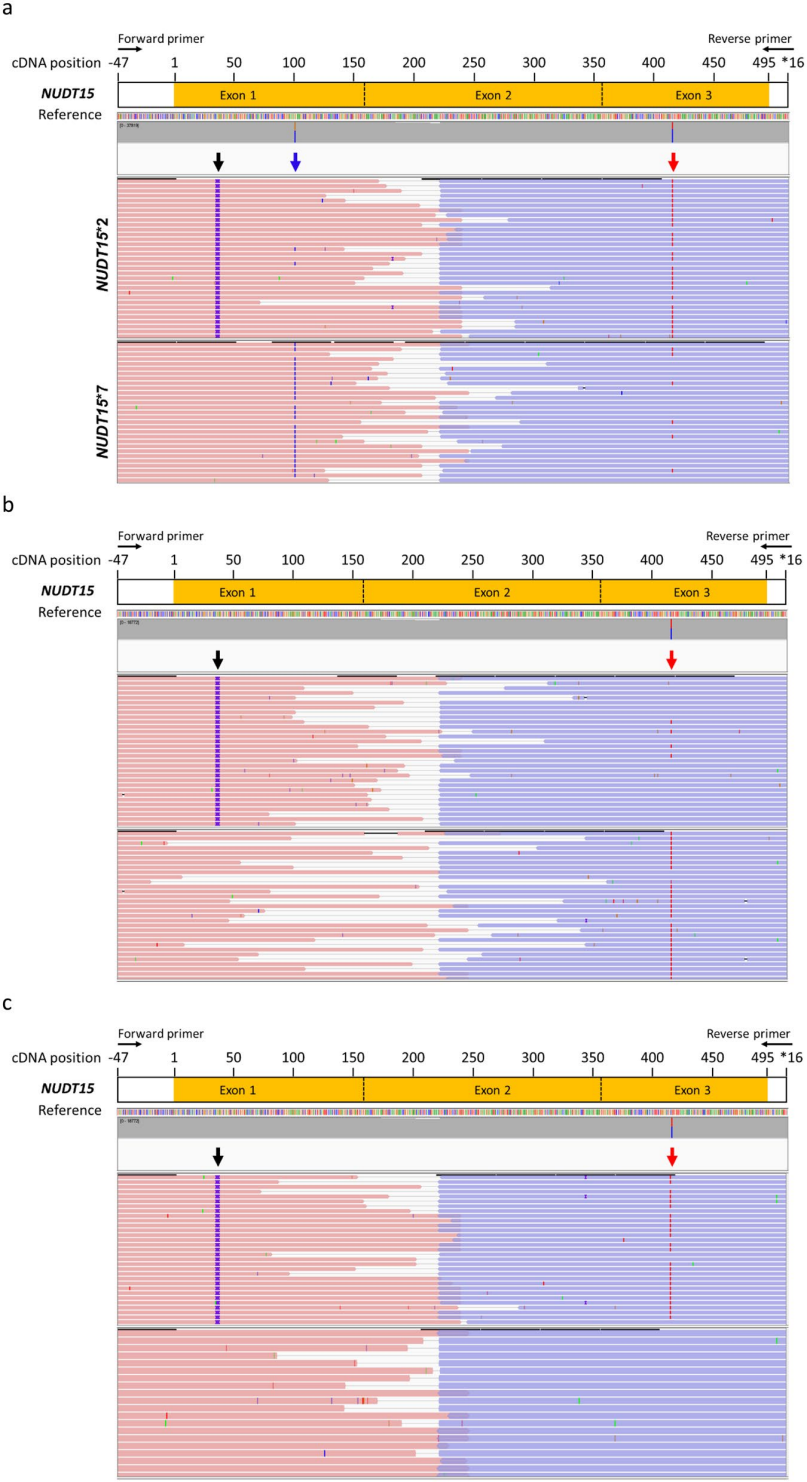
Phased variants of *NUDT15*. (**a**) Patients (NTUCH07) with NUDT15*1/*2, (**b**) patients (NTUCH27) with NUDT15*3/*6, and (**c**) patients (NTUCH37) with NUDT15*2/*7 are shown. Reads were mapped to *NUDT15* mRNA reference sequence (NM_018283), and cDNA position relative to gene structure is presented. The *NUDT15* variants are indicated with arrows, and reads were grouped by haplotype for these variants. Black arrow: c.36_37insGGAGTC; blue arrow: c.101G > C; red arrow: c.415C > T. The paired-end reads are represented, and the reads are colored red for forward strand and blue for reverse strand.

To resolve the diplotypes of patients with multiple heterozygous variants, we sequenced *NUDT15* cDNA by paired-end sequencing. Thirty-seven patients had multiple heterozygous variants. We also included the cDNA of 12 patients with known diplotypes (4 with NUDT15*1/*1, 2 with NUDT15*1/*3, 2 with NUDT15*1/*5, 1 with NUDT15*1/*6, 2 with NUDT15*2/*2, and 1 with NUDT15*2/*3) as experimental controls^30^. The reads were phased, and the results showed that all but two samples could be classified as NUDT15*1/*2 (Fig. 2a). The two samples with different allele combinations were classified as NUDT15*3/*6 (Fig. 2b) and NUDT15*2/*7 (Fig. 2c).

### cDNA sequencing of the NUDT15*3/*6 patient

**Figure 3:**
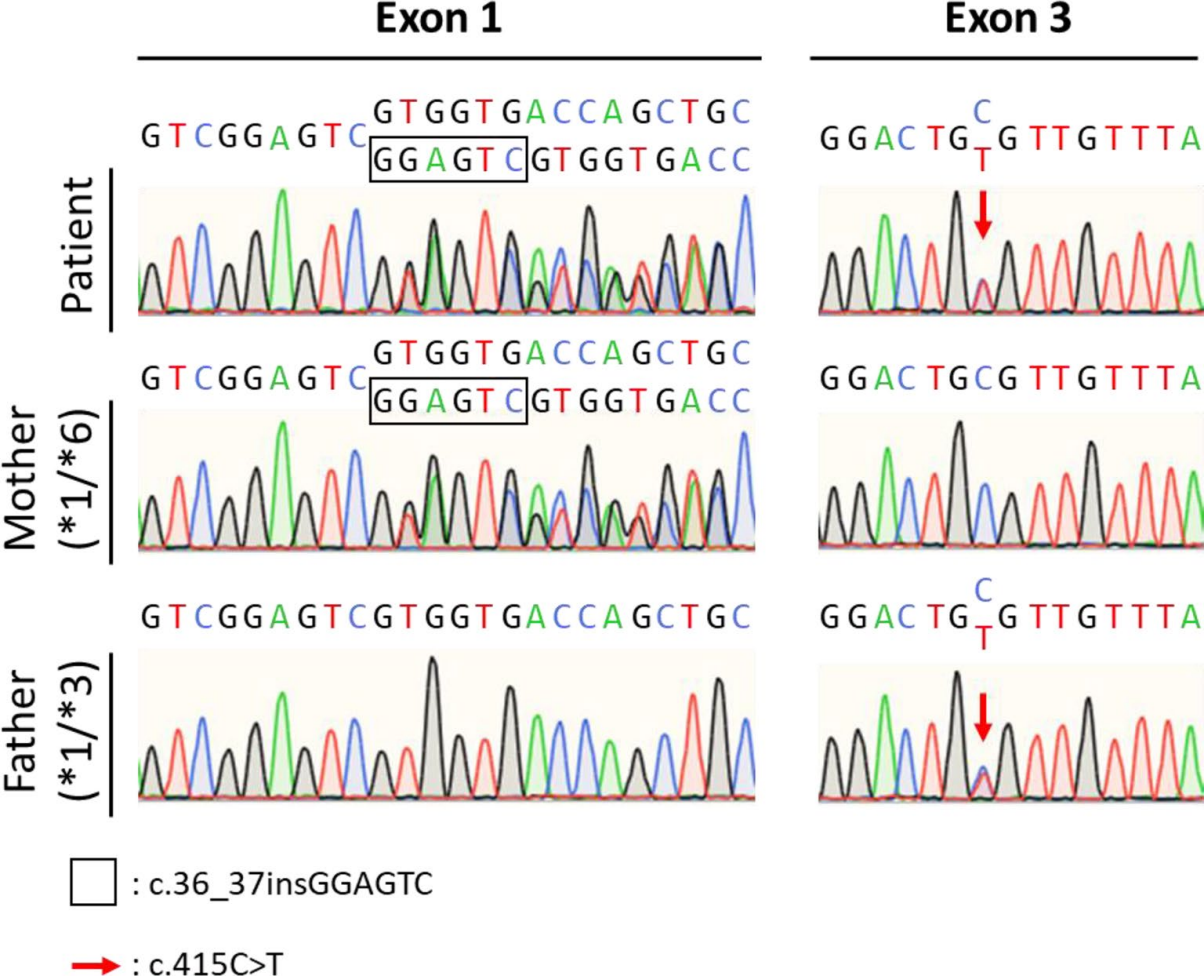
Patient with heterozygous c.36_37insGAGTCG and c.415C > T variants. We performed Sanger sequencing of the biological parents of the patient. The mother had a heterozygous c.36_37insGAGTCG variant (NUDT15*1/*6) and father had a heterozygous c.415C > T variant (NUDT15*1/*3).

To validate the targeted sequencing result for the patient with NUDT15*3/*6, the *NUDT15* cDNA was cloned into a vector, and the haplotypes were resolved by Sanger sequencing of the vector. The results showed that the c.36_37insGGAGTC and c.415C > T alleles were located *in trans*; thus, the genome of this patient was determined to harbor a compound heterozygous NUDT15*3/*6 variant (Figure S1). We also genotyped the biological parents of this patient and found that the genomes of the parents harbored NUDT15*1/*6 and NUDT15*1/*3. The genotyping of the biological parents of patients provides an additional line of evidence to determine the diplotypes (Fig. 3).

### cDNA sequencing of the NUDT15*2/*7 patient

We also validated the result of the patient with NUDT15*2/*7 by cDNA cloning and showed that c.101G > C was located *in trans* with c.36_37insGGAGTC and c.415C > T (Figure S2). The biological parents of the patients were genotyped, and we found that the father was homozygous for c.36_37insGGAGTC and c.415C > T (NUDT15*2/*2), whereas the mother was heterozygous for the c.36_37insGGAGTC, c.415C > T, and c.101G > C variants, which matched the genotype of the patient. Because the patient must have had an NUDT15*2 allele from the father, the c.101G > C allele should have been inherited from the mother; we can posit that the diplotype of the mother is NUDT15*2/*7.

### Tolerated doses of 6-MP among patients with the NUDT15 variant

**Figure 4:**
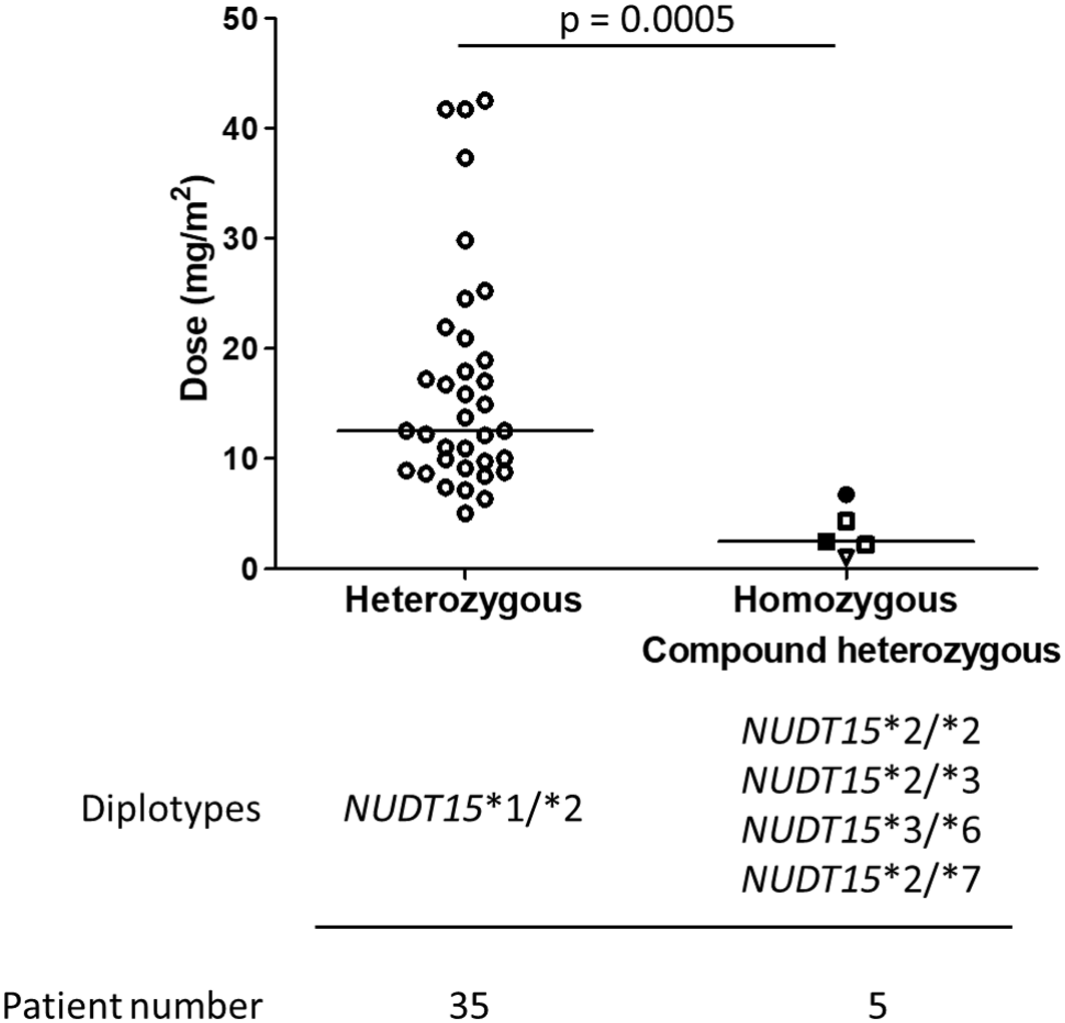
Distribution of tolerated 6-MP doses according to *NUDT15* diplotypes. Heterozygous (open circles: NUDT15*1/*2), homozygous (open triangle: NUDT15*2/*2, open square: NUDT15*2/*3), or compound heterozygous (closed square: NUDT15*3/*6, closed circle: NUDT15*2/*7)) and patient number are represented. The P-value was calculated using Mann–Whitney U test.

We evaluated the median tolerated 6-MP dose in patients with the *NUDT15* variants. The median tolerated 6-MP dose among all 35 patients with the NUDT15*1/*2 genotype was 12.5 mg/m^2^ (5.0–42.5 mg/m^2^), and the doses were extremely low for the patients with NUDT15*2/*2 or NUDT15*2/*3 (1, 2.2, and 4.3 mg/m^2^). The two patients with compound heterozygous variants, NUDT15*3/*6 and NUDT15*2/*7, tolerated only 2.5 and 6.7 mg/m^2^/day mercaptopurine, respectively, and this was similar to the patients with homozygous variants. We observed that patients with homozygous and compound heterozygous *NUDT15* variants tolerated a significantly lower median 6-MP dose than patients with heterozygous variants (Fig. 4).

## Discussion

Our study and that of Tsujimoto et al.^32^ showed that most patients with the *NUDT15* c.36_37insGGAGTC and c.415C > T variants harbored heterozygous NUDT15*1/*2. Tsujimoto et al. defined the diplotypes of 14 patients carrying the two variants (i.e., c.415C > T and c.36_37insGGAGTC) by droplet digital PCR (ddPCR) and restriction enzyme-PCR (RE-PCR)^32^. We developed another method using *NUDT15* cDNA-targeted sequencing. The *NUDT15* cDNA was sequenced by paired-end sequencing using Illumina MiSeq, and our results were confirmed by cloning. Compared with ddPCR and RE-PCR, targeted sequencing of *NUDT15* cDNA can help determine the diplotypes without genotyping information of a patient.

Currently, *NUDT15* genotyping is indicated before mercaptopurine administration for pediatric ALL^31^. Poor metabolizers are defined as individuals carrying two nonfunctioning alleles (NUDT15*2/*2, NUDT15*2/*3, and NUDT15*3/*3). Potential intermediate metabolizers are individuals carrying one allele of uncertain function plus one nonfunctioning allele, such as NUDT15*2/*5 and NUDT15*3/*6^31^. Our patient with the NUDT15*3/*6 combination tolerated only 2.5 mg/m^2^/day mercaptopurine, and this was similar to the dose for poor metabolizers. Another patient with NUDT15*2/*7 could also tolerate very low daily doses of mercaptopurine. The c.101G > C variant of *NUDT15* is also very rare in Asians, and its function was confirmed by Moriyama et al^19^. From our experience with these two patients, any combination of two mutant alleles with known functional roles might be classified as compound heterozygous, and the mercaptopurine dose should be the same as that administered to homozygous patients. Because mercaptopurine is widely administered for other diseases, such as inflammatory bowel disease^33,34^, its clinical significance has also been validated in this subgroup of patients. The identification of these two compound heterozygous patients suggests that testing laboratories should be cautious while interpreting the c.36_37insGGAGTC and c.415C > T variants of *NUDT15* in genetic testing of pediatric patients with ALL taking mercaptopurine or other related drugs, rather than only considering these genotypes.

There was a major limitation to this study. From a cost and benefit perspective, it might be difficult to use cDNA NGS to determine the diplotype of *NUDT15* in clinical practice; moreover, the incidence of compound heterozygous mutations, such as NUDT15*3/*6, is very rare^18^. Sanger sequencing remains the most convenient and cost-effective method for the analysis of genetic variants of *NUDT15*^17,18,20,25,27,28,31,32,35^. However, to improve clinical outcomes and reduce adverse effects in pediatric patients with ALL, appropriate preemptive diagnosis of pharmacogenetic variants might be warranted^31,36,37^. There were other genetic polymorphisms related to some common adverse effects of chemotherapy, such as vincristine neuropathy, asparaginase hypersensitivity, hypertension, and osteonecrosis due to steroid use^37−45^. NGS cDNA targeted therapy might have some advantages, including sequencing multiple genes simultaneously and identifying the diplotype of *NUDT15* variants if complex variants are present. If multiple genetic variants can be sequenced simultaneously, the cost might be reduced for individual SNPs and avoid tedious multiple Sanger sequencing. Although *NUDT15* variants are very rare, identifying compound homozygous patients before the administration of mercaptopurine might largely reduce the initial dose of mercaptopurine to avoid profound marrow suppression. Thus, the infectious adverse effect might be decreased in this small subset of patients.

In conclusion, most c.36_37insGGAGTC and c.415C > T variants in *NUDT15* are located on the same allele and can be classified as monoallelic variants. Although very rare, there are patients with compound heterozygous *NUDT15* variants. Patients with any combination of functionally verified *NUDT15* alleles might be considered to be poor mercaptopurine metabolizers, and these patients should be administered a relatively low dose of this drug. An appropriate definition of compound heterozygous variants in *NUDT15* might require genotyping of the biological parents of the patient or other molecular methods, such as targeted sequencing, ddPCR, RE-PCR, and cloning.

### Ethics declaration

This study was conducted in accordance with the Declaration of Helsinki guidelines. Written informed consent was obtained from all study participants or their guardians. The study was approved by the Institutional Research Board of National Taiwan University Hospital (NTUH IRB number 201510016RIND).

### Consent to participate

None.

## Supplementary information

Supplementary file1

## Data Availability

Data of cDNA sequencing of *NUDT15* have been deposited at https://www.ncbi.nlm.nih.gov/sra/PRJNA655987.
