# Supplementary material for: Determination of NUDT15 variants by targeted sequencing can identify compound heterozygosity in pediatric acute lymphoblastic leukemia patients
Source: Sci Rep. 2020 Sep 1;10:14400. doi: 10.1038/s41598-020-71468-y (PMC7463237; doi:10.1038/s41598-020-71468-y)
Supplement: Supplementary file 1 — Supplementary file1 [file 41598_2020_71468_MOESM1_ESM.pdf]

Determination of *NUDT15* variants by targeted sequencing can identify compound heterozygosity in pediatric acute lymphoblastic leukemia patients

Chih-Hsiang Yu[1], Ya-Hsuan Chang[2], Der-Shiun Wang[3,4], Shiann-Tarng Jou[5], Chien-Yu Lin[2], Kai-Hsin Lin[5], Meng-Yao Lu[5], Lovely Raghav[2], Hsiu-Hao Chang[5], Kang-Hsi Wu[6], Shu-Wei Chou[5], Yu-Ling Ni[7], Dong-Tsamn Lin[5,7], Shu-Wha Lin[1], Hsuan-Yu Chen[2], Yung-Li Yang[5,7]

[1]Departments of Clinical Laboratory Sciences and Medical Biotechnology, National Taiwan University, Taipei, Taiwan

[2]Institute of Statistical Science Academia Sinica, Taipei, Taiwan

[3]Department of Pediatrics, Tri-service General Hospital, Taipei, Taiwan

[4]Graduate Institute of Clinical Medicine, College of Medicine, National Taiwan University, Taipei, Taiwan

[5]Department of Pediatrics, National Taiwan University Hospital and National Taiwan University College of Medicine, Taipei, Taiwan

[6]Division of Pediatric Hematology &Oncology, China Medical University Children's Hospital, Taichung, Taiwan

[7]Department of Laboratory Medicine, National Taiwan University Hospital and National Taiwan University College of Medicine, Taipei, Taiwan

CORRESPONDING AUTHOR

Yung-Li Yang

No. 7, Chung Shan S. Rd, Zhongshan S. Rd, Zhongzheng Dist, Taipei City 10041, Taiwan

Tel.: +886-2-23123456-71712

E-mail: yangyl92@ntu.edu.tw

Supplementary Table 1. Primer sequences

Targets	Primer sequence (5′ to 3′)
**cDNA cloning**	
NUDT15 cDNA_Forward	GTGAGCGCGTCACTTCCTGC
NUDT15 cDNA_ Reverse	ATCAAATCTTCTCGGCCACCTAGAG
**Barcoded primer for targeted-sequencing of cDNA**	
F_D701	ATTACTCGGTGAGCGCGTCACTTCCTGC
F_D702	TCCGGAGAGTGAGCGCGTCACTTCCTGC
F_D703	CGCTCATTGTGAGCGCGTCACTTCCTGC
F_D704	GAGATTCCGTGAGCGCGTCACTTCCTGC
F_D705	ATTCAGAAGTGAGCGCGTCACTTCCTGC
F_D706	GAATTCGTGTGAGCGCGTCACTTCCTGC
F_D707	CTGAAGCTGTGAGCGCGTCACTTCCTGC
R_D501	TATAGCCTATCAAATCTTCTCGGCCACCTAGAG
R_D502	ATAGAGGCATCAAATCTTCTCGGCCACCTAGAG
R_D503	CCTATCCTATCAAATCTTCTCGGCCACCTAGAG
R_D504	GGCTCTGAATCAAATCTTCTCGGCCACCTAGAG
R_D505	AGGCGAAGATCAAATCTTCTCGGCCACCTAGAG
R_D506	TAATCTTAATCAAATCTTCTCGGCCACCTAGAG
R_D507	CAGGACGTATCAAATCTTCTCGGCCACCTAGAG
R_D508	GTACTGACATCAAATCTTCTCGGCCACCTAGAG

Supplementary Table 2. The detailed information for patients selected for haplotyping using targeted sequencing

Patient ID	6-MP dose (mg/m^2)	Genotyping by Sanger sequencing			Phasing result	Primer for targeted sequencing of *NUDT15* cDNA
		Exon 1	Exon 2	Exon 3		
NTUCH01	6.3	het, c.36_37insGGAGTC	wild-type	het, c.415C>T	*NUDT15**1/*2	F_D701+R_D501
NTUCH02	29.8	het, c.36_37insGGAGTC	wild-type	het, c.415C>T	*NUDT15**1/*2	F_D701+R_D502
NTUCH03	20.9	het, c.36_37insGGAGTC	wild-type	het, c.415C>T	*NUDT15**1/*2	F_D701+R_D503
NTUCH04	17.9	het, c.36_37insGGAGTC	wild-type	het, c.415C>T	*NUDT15**1/*2	F_D701+R_D504
NTUCH05	12.5	het, c.36_37insGGAGTC	wild-type	het, c.415C>T	*NUDT15**1/*2	F_D701+R_D505
NTUCH06	9.9	het, c.36_37insGGAGTC	wild-type	het, c.415C>T	*NUDT15**1/*2	F_D701+R_D506
NTUCH07	25.2	het, c.36_37insGGAGTC	wild-type	het, c.415C>T	*NUDT15**1/*2	F_D701+R_D507
NTUCH08	17.2	het, c.36_37insGGAGTC	wild-type	het, c.415C>T	*NUDT15**1/*2	F_D701+R_D508
NTUCH09	41.7	het, c.36_37insGGAGTC	wild-type	het, c.415C>T	*NUDT15**1/*2	F_D702+R_D501
NTUCH10	41.7	het, c.36_37insGGAGTC	wild-type	het, c.415C>T	*NUDT15**1/*2	F_D702+R_D502
NTUCH11	37.3	het, c.36_37insGGAGTC	wild-type	het, c.415C>T	*NUDT15**1/*2	F_D702+R_D503
NTUCH12	2.2	homo, c.36_37insGGAGTC	wild-type	homo, c.415C>T	*NUDT15**2/*2	F_D702+R_D504
NTUCH13	18.9	het, c.36_37insGGAGTC	wild-type	het, c.415C>T	*NUDT15**1/*2	F_D702+R_D505
NTUCH14	24.5	het, c.36_37insGGAGTC	wild-type	het, c.415C>T	*NUDT15**1/*2	F_D702+R_D506
NTUCH15	14.9	het, c.36_37insGGAGTC	wild-type	het, c.415C>T	*NUDT15**1/*2	F_D702+R_D507
NTUCH16	42.5	het, c.36_37insGGAGTC	wild-type	het, c.415C>T	*NUDT15**1/*2	F_D702+R_D508
NTUCH17	9.1	het, c.36_37insGGAGTC	wild-type	het, c.415C>T	*NUDT15**1/*2	F_D703+R_D501
NTUCH18	7.1	het, c.36_37insGGAGTC	wild-type	het, c.415C>T	*NUDT15**1/*2	F_D703+R_D502
NTUCH19	21.9	het, c.36_37insGGAGTC	wild-type	het, c.415C>T	*NUDT15**1/*2	F_D703+R_D503
NTUCH20	12.5	het, c.36_37insGGAGTC	wild-type	het, c.415C>T	*NUDT15**1/*2	F_D703+R_D504
NTUCH21	8.4	het, c.36_37insGGAGTC	wild-type	het, c.415C>T	*NUDT15**1/*2	F_D703+R_D505
NTUCH22	12.1	het, c.36_37insGGAGTC	wild-type	het, c.415C>T	*NUDT15**1/*2	F_D703+R_D506
NTUCH23	15.8	het, c.36_37insGGAGTC	wild-type	het, c.415C>T	*NUDT15**1/*2	F_D703+R_D507
NTUCH24	8.75	het, c.36_37insGGAGTC	wild-type	het, c.415C>T	*NUDT15**1/*2	F_D703+R_D508
NTUCH25	7	het, c.52G>A	wild-type	wild-type	*NUDT15**1/*5	F_D704+R_D501

NTUCH26	10	het, c.36_37insGGAGTC	wild-type	het, c.415C>T	*NUDT15**1/*2	F_D704+R_D502
NTUCH27	2.5	het, c.36_37insGGAGTC	wild-type	het, c.415C>T	*NUDT15**1/*2	F_D704+R_D503
NTUCH28	10.9	het, c.36_37insGGAGTC	wild-type	het, c.415C>T	*NUDT15**1/*2	F_D704+R_D504
NTUCH29	13.7	het, c.36_37insGGAGTC	wild-type	het, c.415C>T	*NUDT15**1/*2	F_D704+R_D505
NTUCH30	17	het, c.36_37insGGAGTC	wild-type	het, c.415C>T	*NUDT15**1/*2	F_D704+R_D506
NTUCH31	11	het, c.36_37insGGAGTC	wild-type	het, c.415C>T	*NUDT15**1/*2	F_D704+R_D507
NTUCH32	12.2	het, c.36_37insGGAGTC	wild-type	het, c.415C>T	*NUDT15**1/*2	F_D704+R_D508
NTUCH33	16.7	het, c.36_37insGGAGTC	wild-type	het, c.415C>T	*NUDT15**1/*2	F_D705+R_D501
NTUCH34	8.62	het, c.36_37insGGAGTC	wild-type	het, c.415C>T	*NUDT15**1/*2	F_D705+R_D502
NTUCH35	5	het, c.36_37insGGAGTC	wild-type	het, c.415C>T	*NUDT15**1/*2	F_D705+R_D503
NTUCH36	9.7	het, c.36_37insGGAGTC	wild-type	het, c.415C>T	*NUDT15**1/*2	F_D705+R_D504
NTUCH37	6.72	het, c.36_37insGGAGTC<br>het, c.101G>C	wild-type	het, c.415C>T	*NUDT15**2/*7	F_D705+R_D505
NTUCH39	1	het, c.36_37insGGAGTC	wild-type	homo, c.415C>T	*NUDT15**2/*3	F_D705+R_D507
NTUCH40	4.3	homo, c.36_37insGGAGTC	wild-type	homo, c.415C>T	*NUDT15**2/*2	F_D705+R_D508
NTUCH41	7.35	wild-type	wild-type	wild-type	*NUDT15**1/*1	F_D706+R_D501
NTUCH42	8.23	wild-type	wild-type	wild-type	*NUDT15**1/*1	F_D706+R_D502
NTUCH43	5.7	wild-type	wild-type	wild-type	*NUDT15**1/*1	F_D706+R_D503
NTUCH44	8.9	wild-type	wild-type	wild-type	*NUDT15**1/*1	F_D706+R_D504
NTUCH45	1.81	wild-type	wild-type	het, c.415C>T	*NUDT15**1/*3	F_D706+R_D505
NTUCH46	8.6	wild-type	wild-type	het, c.415C>T	*NUDT15**1/*3	F_D706+R_D506
NTUCH47	2.55	het, c.52G>A	wild-type	wild-type	*NUDT15**1/*5	F_D706+R_D507
NTUCH48	1	het, c.36_37insGGAGTC	wild-type	wild-type	*NUDT15**1/*6	F_D706+R_D508
NTUCH49	7.35	het, c.36_37insGGAGTC	wild-type	het, c.415C>T	*NUDT15**1/*2	F_D707+R_D501
NTUCH50	8.9	het, c.36_37insGGAGTC	wild-type	het, c.415C>T	*NUDT15**1/*2	F_D707+R_D502

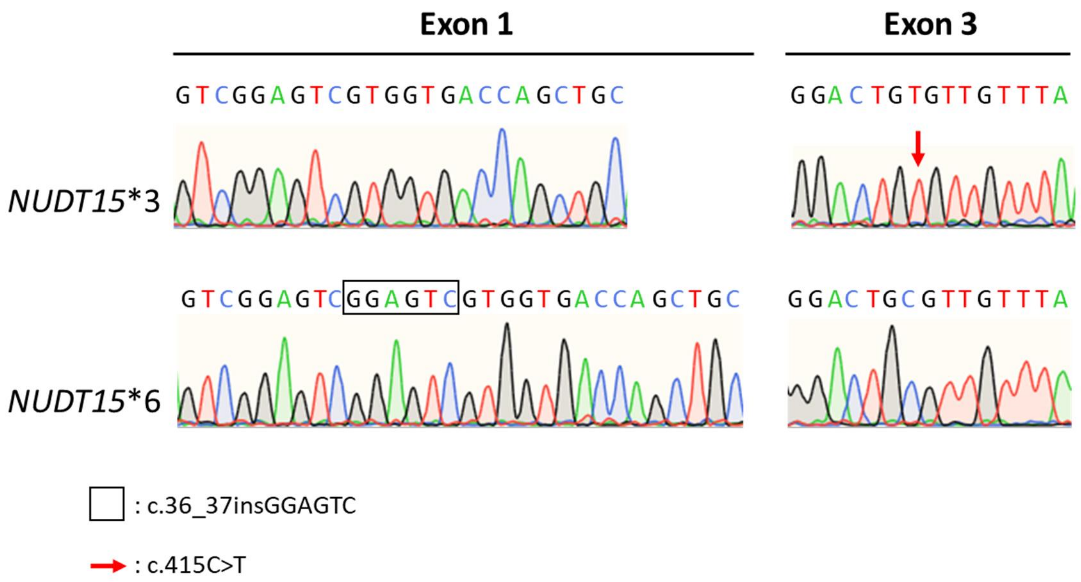

Supplementary Figure 1. cDNA cloning showed that there are two different types of *NUDT15* alleles. One is *NUDT15**3, and the other is *NUDT15**6.

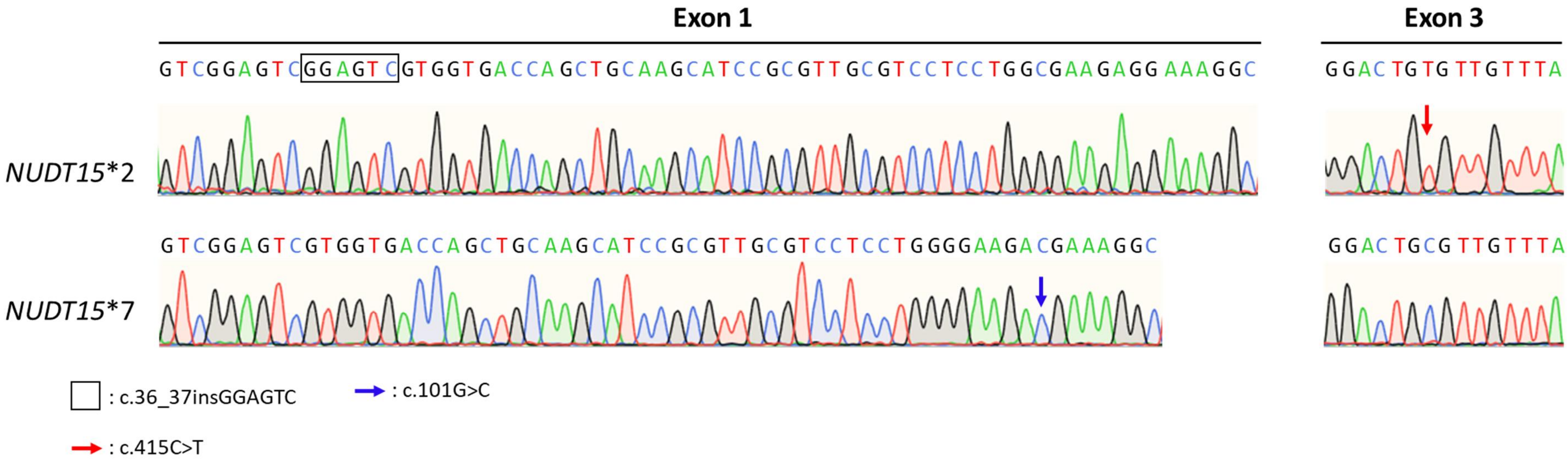

Supplementary Figure 2. cDNA cloning showed that there are two different types of *NUDT15* alleles. One allele is *NUDT15**2, and the other allele is *NUDT15**7.

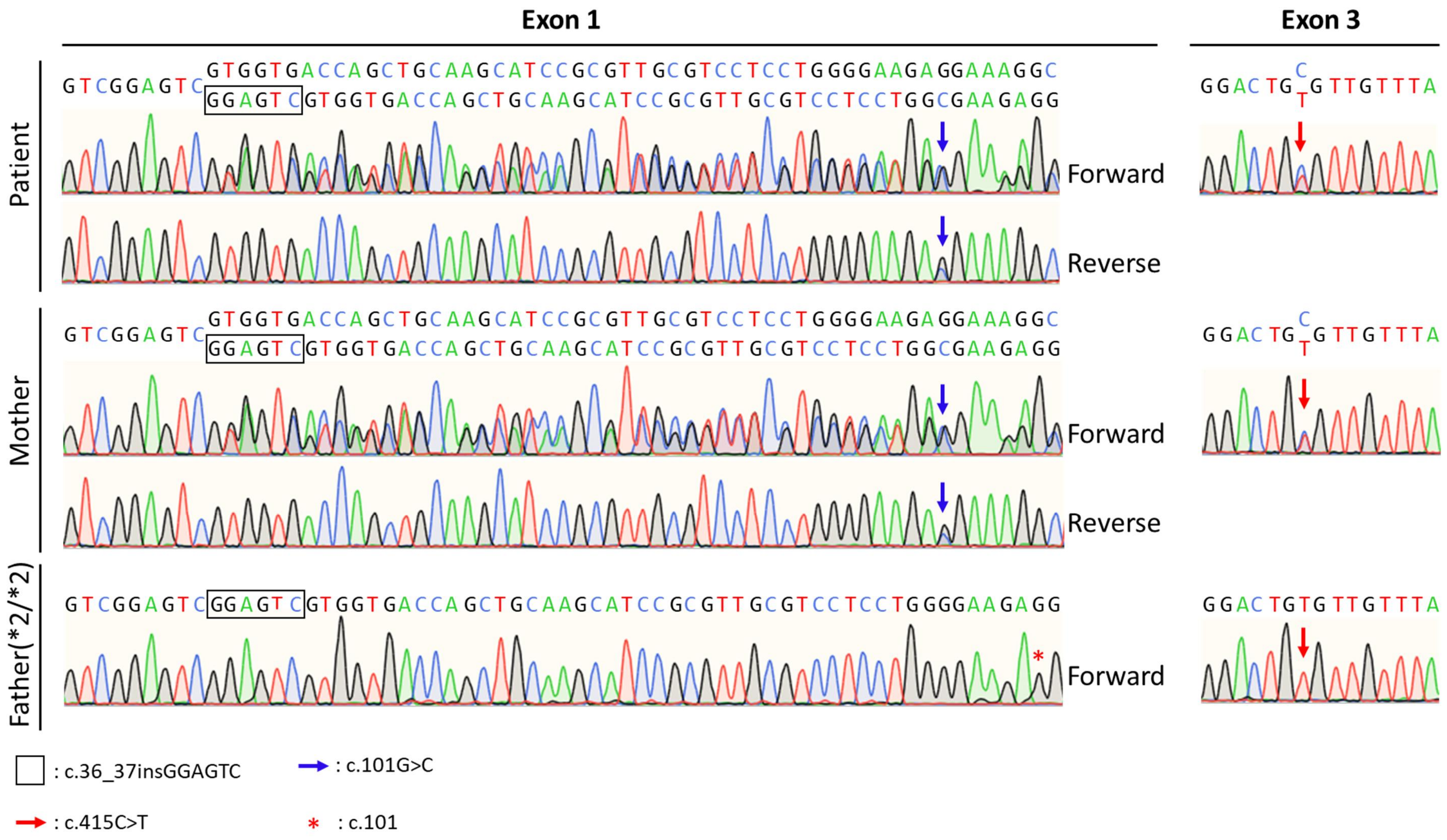

Supplementary Figure 3. A patient had heterozygous c.36_37insGGAGTC, c.101G>C, and c.415C>T variants. We also performed Sanger sequencing of his biological parents. His mother had heterozygous c.36_37insGGAGTC, c.101G>C, and c.415C>T variants, and her father had homozygous c.36_37insGGAGTC and c.415C>T (*NUDT15**2/*2) variants.
